# To Recognize the Use of International Standards for Making Harmonized Regulation of Medical Devices in Asia-Pacific

**DOI:** 10.4103/0975-1483.66804

**Published:** 2010

**Authors:** K Anand, KS Saini, Y Chopra, SK Binod

**Affiliations:** *Department of Pharmaceutical Sciences, Jodhpur National University, Jodhpur, Rajasthan, India*; 1 *Medical Affairs, Johnson and Johnson Medical, Division of Johnson and Johnson Ltd., Gurgaon, Haryana, India*; 2 *Kumaun University, Bhimtal Campus, Nainital, Uttarakhand, India*

**Keywords:** Essential principles, harmonization, international organization, recognized standards, regulatory system

## Abstract

‘Medical Devices’ include everything from highly sophisticated, computerized, medical equipment, right down to simple wooden tongue depressors. Regulations embody the public expectations for how buildings and facilities are expected to perform and as such represent public policy. Regulators, who develop and enforce regulations, are empowered to act in the public’s interest to set this policy and are ultimately responsible to the public in this regard. Standardization contributes to the basic infrastructure that underpins society including health and environment, while promoting sustainability and good regulatory practice. The international organizations that produce International Standards are the International Electrotechnical Commission (IEC), the International Organization for Standardization (ISO), and the International Telecommunication Union (ITU). With the increasing globalization of markets, International Standards (as opposed to regional or national standards) have become critical to the trading process, ensuring a level playing field for exports, and ensuring that imports meet the internationally recognized levels of performance and safety. The development of standards is done in response to sectors and stakeholders that express a clearly established need for them. An industry sector or other stakeholder group typically communicates its requirement for standards to one of the national members. To be accepted for development, a proposed work item must receive a majority support of the participating members, who verify the global relevance of the proposed item. The regulatory authority (RA) should provide a method for the recognition of international voluntary standards and for public notification of such recognition. The process of recognition may vary from country to country. Recognition may occur by periodic publication of lists of standards that a regulatory authority has found will meet the Essential Principles. In conclusion, International standards, such as, basic standards, group standards, and product standards, are a tool for harmonizing regulatory processes, to assure the safety, quality, and performance of medical devices. Standards represent the opinion of experts from all interested parties, including industry, regulators, users, and others.

## INTRODUCTION

The term ‘Medical Devices’ includes everything from highly sophisticated, computerized, medical equipment, right down to simple wooden tongue depressors. Medical devices have extended the ability of physicians to diagnose and treat diseases, making great contributions to health and quality of life, and without any doubt, these technologies have changed the mainstream practice of medicine. The intended primary mode of action of a medical device on the human body, in contrast to that of medicinal products, is not metabolic, immunological, or pharmacological. These devices include not only diagnostic technology but also analytical techniques, using high-resolution chromatography, polymerase chain reaction (PCR), and monoclonal antibodies, providing physicians with new, accurate, and rapid information. On the therapeutic side, devices save lives and improve the quality of life. For example, dialysis therapy extends lives for end-stage renal disease patients, orthopedic implants enable patients to walk again, and minimally invasive technologies allow surgeries that are safer, with less pain and trauma, requiring significantly shorter hospital stays.[1] The medical device market is consolidating to respond to market needs. Consolidation will promote accelerated time in the market, greater returns on invested capital, and transformational innovation, while maintaining high quality and patient efficacy. In accordance, therefore, medical devices and equipments are set for a vibrant growth in the near future. However, overall, in a broad sense, we can describe Medical Devices as any instrument, apparatus, implement, machine, appliance, implant, *in vitro* reagent or calibrator, software, material or other similar or related article, intended by the manufacturer to be used, alone or in combination, for human beings, for one or more of the specific purposes of:

- Diagnosis, prevention, monitoring, treatment or alleviation of disease
- Diagnosis, monitoring, treatment, alleviation of or compensation for an injury
- Investigation, replacement, modification, or support of the anatomy or of a physiological process
- Supporting or sustaining life
- Control of conception
- Disinfection of medical devices
- Providing information for medical purposes by means of *in vitro* examination of specimens derived from the human body, and that which does not achieve its primary intended action in or on the human body by pharmacological, immunological or metabolic means, but which may be assisted in its function by such means.[2]

What does Medical Device Harmonization actually mean?

*Medical device harmonization*: “To encourage convergence in regulatory practices related to ensuring the safety, effectiveness / performance, and quality of medical devices, promoting technological innovation and facilitating international trade”.[3]

*Harmonization*: “To bring into Agreement or Harmony”

Harmony*: “Agreement in Feeling or Opinion”

(*According to American Heritage College dictionary, third edition; 2000)

Building regulatory systems consists of regulations adopted into law, through whatever legislative or administrative procedures that are appropriate to the legal system in place, supported by standards that provide the details on what is considered necessary or sufficient to be considered for compliance. Regulations embody the public expectations for how buildings and facilities are expected to perform and as such represent public policy. Regulators, who develop and enforce regulations, are empowered to act in the public interest, to set this policy, and are ultimately responsible to the public in this regard.[4]

Standards can provide details of methods and evaluation criteria too complex to include within the regulations themselves. Standards, as more technical documents, rely on significant input from technical experts and are often developed by private groups who may have financial interests in the items covered. Standards employed in regulatory contexts should be developed in a fair and open manner and many countries have mechanisms in place to ensure that standards do not restrain trade or limit competition. The ultimate goal of standardization is to achieve an international accord on all technical matters relating to the exchange of goods and services between one nation and another.

International Standards and their use in technical regulations on products, production methods, and services play an important role in sustainable development and trade facilitation through the promotion of safety, quality, and technical compatibility. The benefits derived are significant. Standardization contributes to the basic infrastructure that underpins society including health and environment, while promoting sustainability and good regulatory practice.

The international organizations that produce International Standards are the International Electrotechnical Commission (IEC), the International Organization for Standardization (ISO), and the International Telecommunication Union (ITU). IEC covers electrotechnology and related conformity assessment, ITU covers telecommunications, and ISO covers nearly all other technical fields, a number of service sectors, management systems, and conformity assessment.[4] International Standards or national or regional adoption of International Standards, assist in the operation of domestic markets, and also increase competitiveness and provide an excellent source of technology transfer. They play an integral role in the protection of consumers and the environment.

With the increasing globalization of markets, International Standards (as opposed to regional or national standards) have become critical to the trading process, ensuring a level playing field for exports, and ensuring that imports meet internationally recognized levels of performance and safety.

International standards can be broadly sub-divided into three categories, namely product, process, and management system standards. The first refers to the characteristics related to quality and safety for example. Process standards refer to the conditions under which products and services are to be produced, packaged or refined. Management system standards assist organizations to manage their operations. They are often used to help create a framework, which then allows the organization to consistently achieve the requirements that are set out in the product and process standards.[4]

International standards can be used to support and simplify the process of development and application of technical regulations. The advantages of doing this are listed herewith:[5]

- They support the technical aspects of societal and environmental policies and contribute to sustainable development across the world
- They offer the same level of consumer protection whether applied in a mature or an evolving economy
- They allow products to be supplied and used across different markets, facilitating regulatory compliance and enhancing the market access opportunities for small enterprises
- They reflect the state-of-the-art and serve as a vehicle for the dissemination of new technologies and innovative practices
- They can become national standards after a national public enquiry process is carried out by a country’s standards body, which can reduce the need for the regulator to hold national consultations
- They can be used as a basis for national technical regulations without causing unnecessary technical barriers to trade
- They offer a complete range of tools for various modes of conformity assessment
- They are used for conformity assessment, to enhance confidence in products, systems, processes, services or personnel
- They are developed using procedures that ensure that the thousands of standards available avoid duplication and conflict with each other

## DEVELOPMENT OF INTERNATIONAL STANDARDS [FIGURE 1]

The development of standards is conducted in response to sectors and stakeholders that express a clearly established need for them. An industry sector or other stakeholder group typically communicates its requirement for standards to one of the national members. To be accepted for development, a proposed work item must receive the majority support of the participating members, which verifies the global relevance of the proposed item.

**Figure 1:**
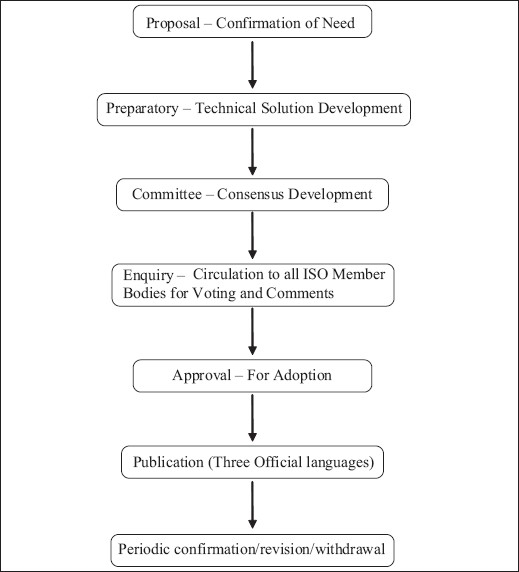
Stages of development of international standards

To summarize, we can say that the responsible persons for the development of standards are:[6]

- Experts from industrial, technical, and business sectors
- Representatives of Government agencies, testing laboratories, consumer associations, non-governmental organizations, and academia
- Those that participate as a national delegation, chosen by the national member body.

To achieve harmonization, the following principles are recommended:[7]

- The regulatory authority and industry should encourage, support, and contribute to the development of international standards that may be useful in demonstrating conformity of medical devices with the Essential Principles
- The regulatory authority should encourage the use of international standards
- The regulatory authority should establish a mechanism for recognizing international standards, to provide manufacturers with a method of demonstrating conformity with the Essential Principles. This mechanism should also include a procedure for withdrawal of recognition. Preference should be given to standards developed in accordance with principles of procedural transparency and rules that require public comment, periodic revisions, and the consideration and resolution of all public comments
- If a manufacturer chooses not to apply to a recognized standard in part or in full, then this is acceptable if conformity with the Essential Principles can be demonstrated by other means
- It is preferable, for harmonization purposes, to use international standards, but the Regulatory Authority should also be prepared to accept the use by manufacturers of global, national, regional or industry standards as a means of demonstrating conformity
- Standards Bodies developing or revising standards of use with medical devices, should consider the suitability of such standards for demonstrating conformity with the Essential Principles and should identify which of the Essential Principles they satisfy

Standards should represent the generally acknowledged state of technology and practice. However, preference for the use of recognized standards should not discourage the use of new technologies. Not all devices or elements of safety and / or performance may be addressed by recognized standards, especially for new types of devices and emerging technologies

## RECOGNITION OF STANDARDS

The regulatory authority should provide a method for the recognition of international voluntary standards and for public notification of such recognition. The process of recognition may vary from country to country. The method should include a mechanism of periodic review and realignment of nationally recognized standards to the international standards. Recognition may occur by periodic publication of lists of standards that a regulatory authority has found will meet the Essential Principles. Persons intending to market medical devices should obtain information from the relevant regulatory authority, through official publications, on the recognized standards.[7]

Conformity with recognized standards may be used by the manufacturer to demonstrate conformity with the relevant Essential Principles and / or specific premarket or post-market requirements of the regulatory authority. When used, the manufacturer should identify the version and date of the relevant recognized standard(s) in the technical documentation.

### Revision of recognized standards

A revision of recognized standards may occur, for example, in the following circumstances:

- A requirement in a specific standard is determined to be inadequate to ensure conformity to a specific Essential Principle
- One or more of the Essential Principles has changed
- Changes in the state of technology or accepted practice necessitate revising the technical specifications in the standard

### Changes to the recognition status

The regulatory authority may cease to recognize a standard for various reasons, such as:

- Safety concerns identified through post-market surveillance activities or user experience
- The availability of a revised version of the standard

Where the regulatory authority considers that for safety reasons a recognized standard ceases to give a presumption of conformity to the Essential Principles, and a revised version has yet to become available, it should fix a date, which may be immediate, after which the standard will no longer give a presumption of conformity to the Essential Principle(s), and publish the withdrawal of recognition in accordance with its procedures for public notification of recognition of standards.[5]

When withdrawing recognition of a standard for reasons other than safety, the regulatory authority should fix a date after which the standard will no longer give a presumption of conformity to the Essential Principle(s). When setting such a date, the RA should establish a transition period that should be adequate to allow manufacturers to respond in an appropriate manner. In such circumstances, the transition period should normally be in the order of three years. Depending upon the extent and nature of the revision, this transition period may be adapted, as appropriate. The RA should publish this information in accordance with its procedures for public notification of the recognition of standards.

### Use of recognized standards when designing and supplying medical devices both during and after the transition period

During the transition period both the prior and the revised version of the recognized standard give presumption of conformity with the Essential Principles, consequently either may be used when designing or supplying medical devices to the end user.

After the transition period, only the revised version of the recognized standard gives presumption of conformity with the relevant Essential Principles. However, manufacturers may choose to use the superseded version of the recognized standard despite the loss of the presumption of conformity to the Essential Principles and may continue to design and / or supply medical devices to the end user according to the superseded standard. In such cases, in order to demonstrate that the medical device conforms to all relevant Essential Principles, the manufacturer is required to justify his decision to use the superseded standard through documented risk assessment, and take any risk mitigation action, as appropriate. The manufacturer’s decision may be subject to review by the Regulatory Authority / Conformity Assessment Body.

### Status of devices designed using the superseded version of the recognized standard and supplied before the end of the transition period

Where a medical device is designed according to the superseded version of the recognized standard, to demonstrate conformity with one or more relevant Essential Principles, and is either in the distribution chain or has been supplied to the end user before the end of the transition period, the manufacturer is not required to take any action unless there are safety implications, in which case the manufacturer should implement a risk mitigation strategy and take appropriate action to address these safety concerns.

### Alternatives to recognized standards

The use of standards is voluntary. Manufacturers should have the option to select alternative solutions to demonstrate that their medical device meets the relevant Essential Principles. Manufacturers may use the ’non-recognized’ standards, in whole or in part, or other methods. Alternative means of demonstrating conformity with the Essential Principles may include:[5]

- National and international standards that have not been given the status of a ‘recognized standard’ by the Regulatory Authority
- Industry agreed methods
- Internal manufacturer standard operating procedures developed by an individual manufacturer
- Other sources that describe the current state of technology and practice related to performance, material, design, methods, processes or practices

The acceptability of such other solutions should be justified and may be subject to review by the Regulatory Authority/ Conformity Assessment Body, as appropriate[7]

## CONCLUSION

Standards, as more technical documents, rely on significant input from technical experts and are often developed by private groups who may have financial interests in the items covered. Standards employed in regulatory contexts should be developed in a fair and open manner and have mechanisms in place to ensure that standards do not restrain trade or limit competition. The ultimate goal of standardization is to achieve international accord on all technical matters relating to the exchange of goods and services between one nation and another. International Standards or national or regional adoptions of International Standards, assist in the operation of domestic markets, and also increase competitiveness and provide an excellent source of technology transfer. They play an integral role in the protection of consumers and the environment. In conclusion, International standards, such as, basic standards, group standards, and product standards, are a tool for harmonizing regulatory processes to assure the safety, quality, and performance of medical devices. Standards represent the opinion of experts from all interested parties, including industry, regulators, users, and others.
